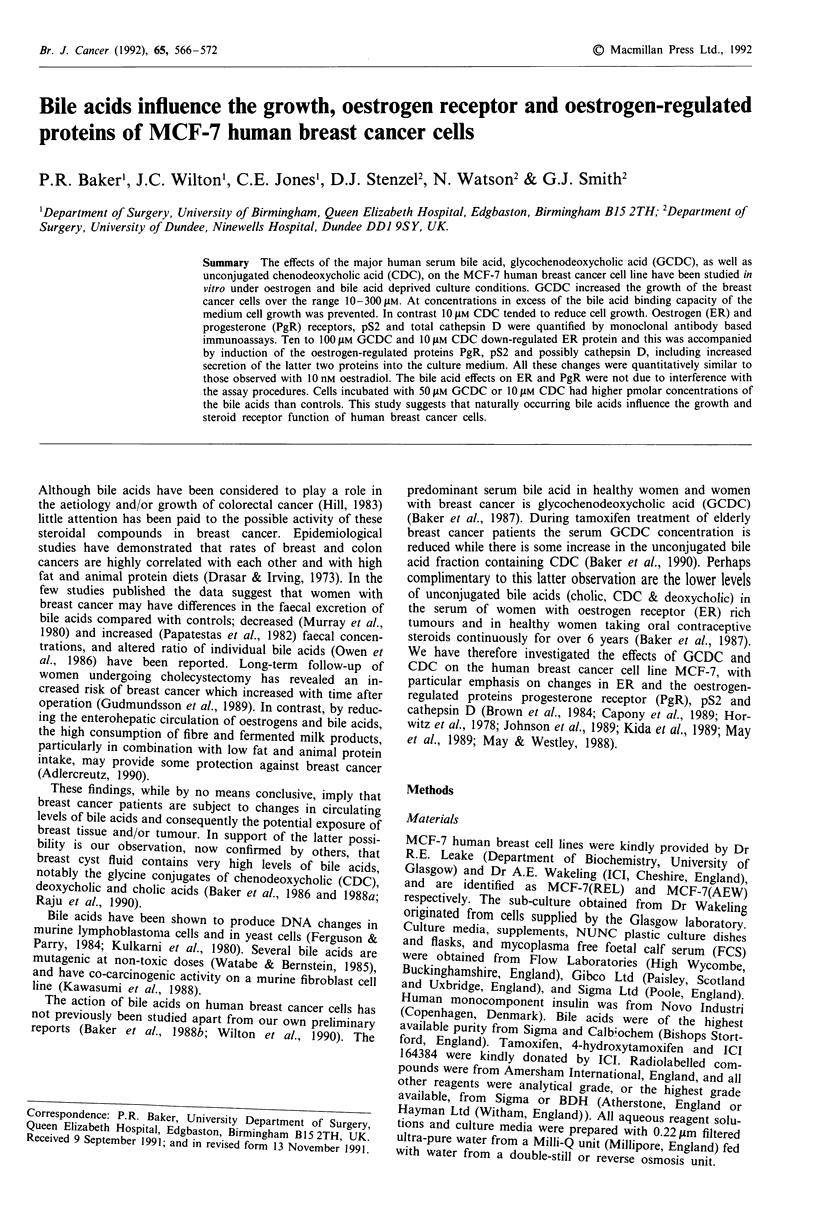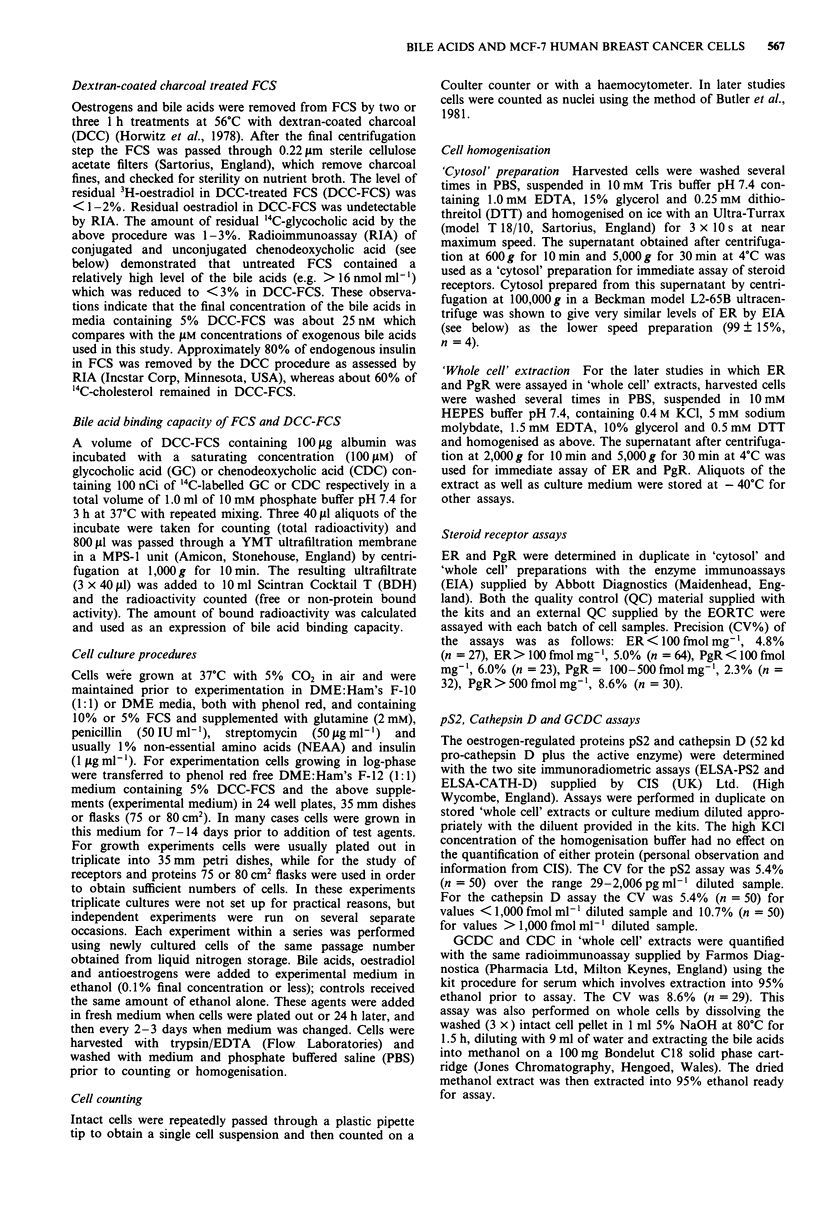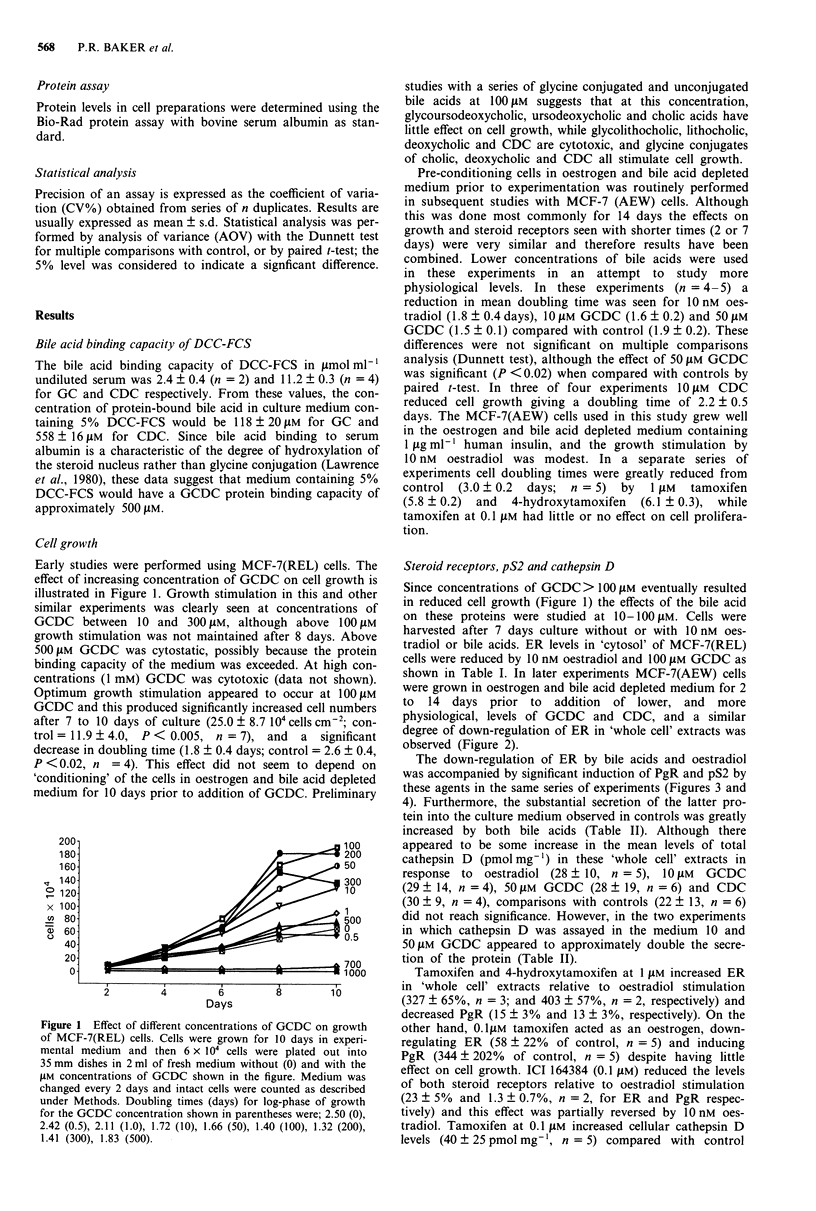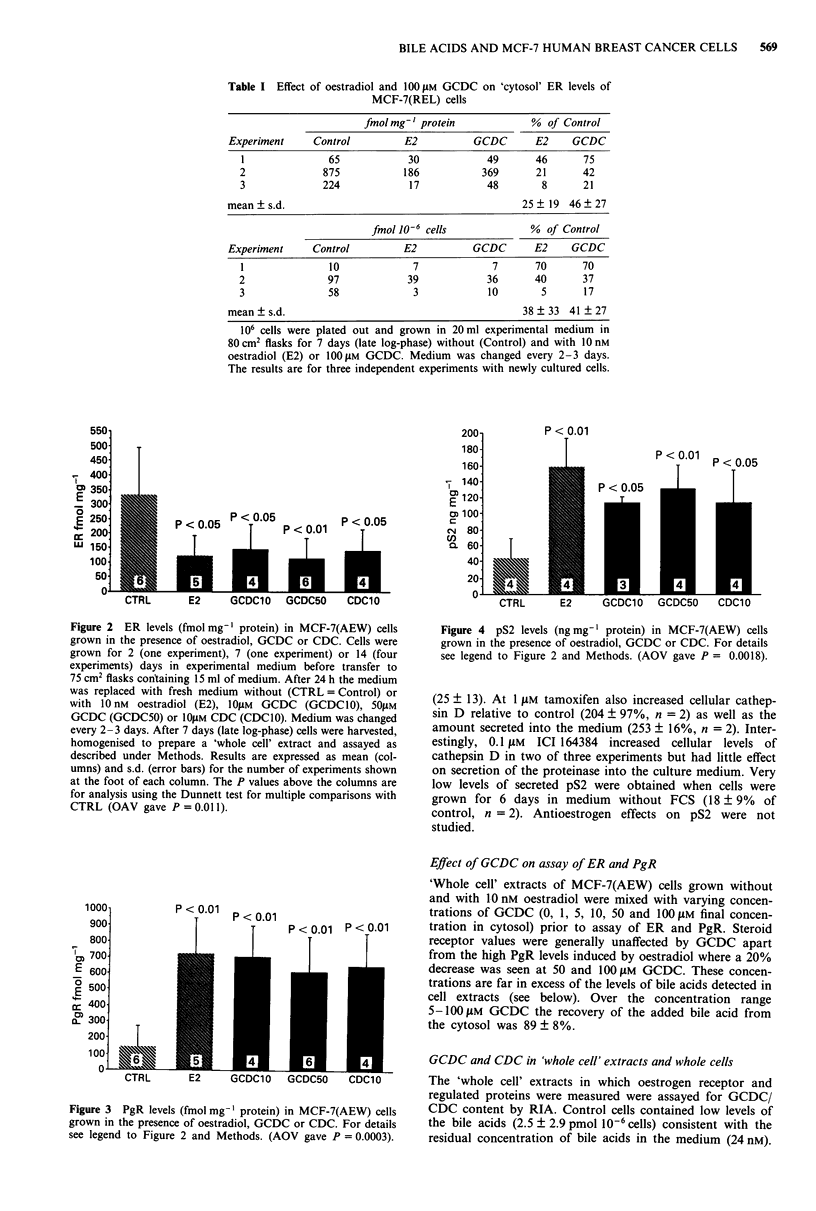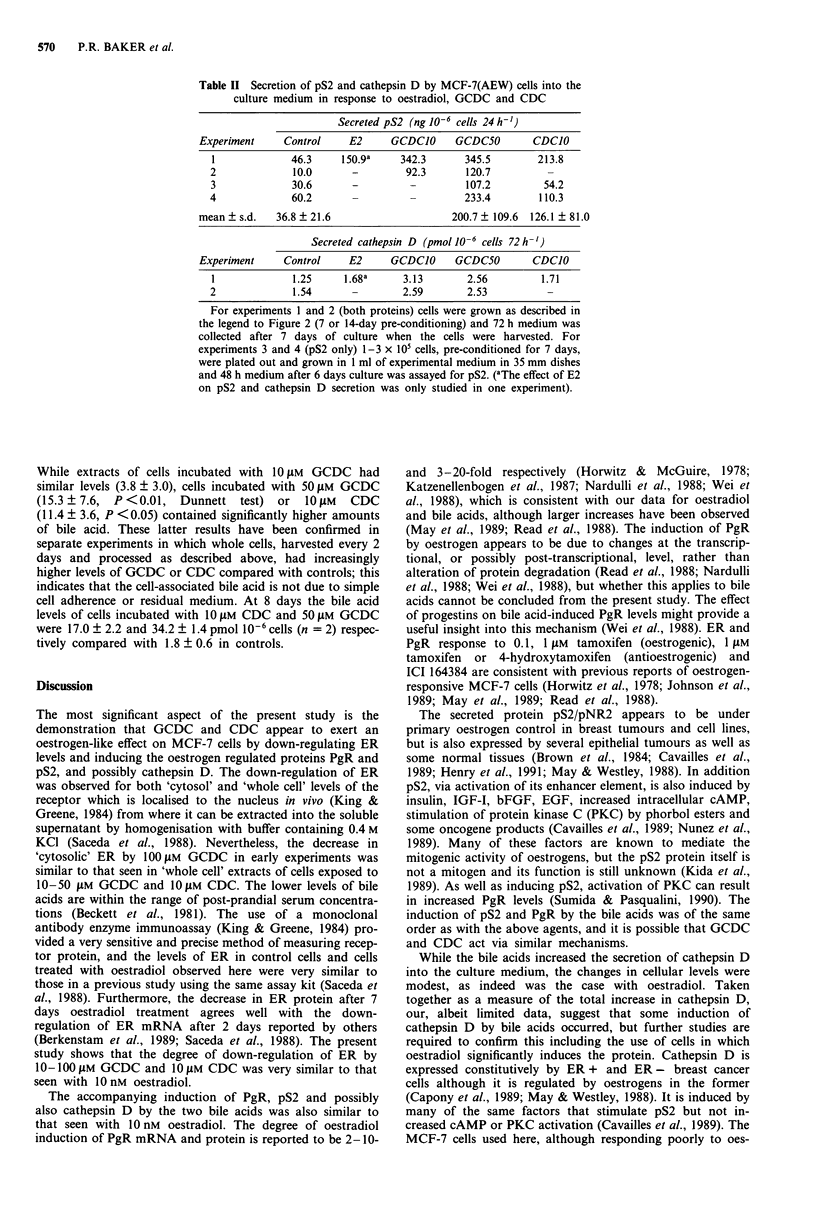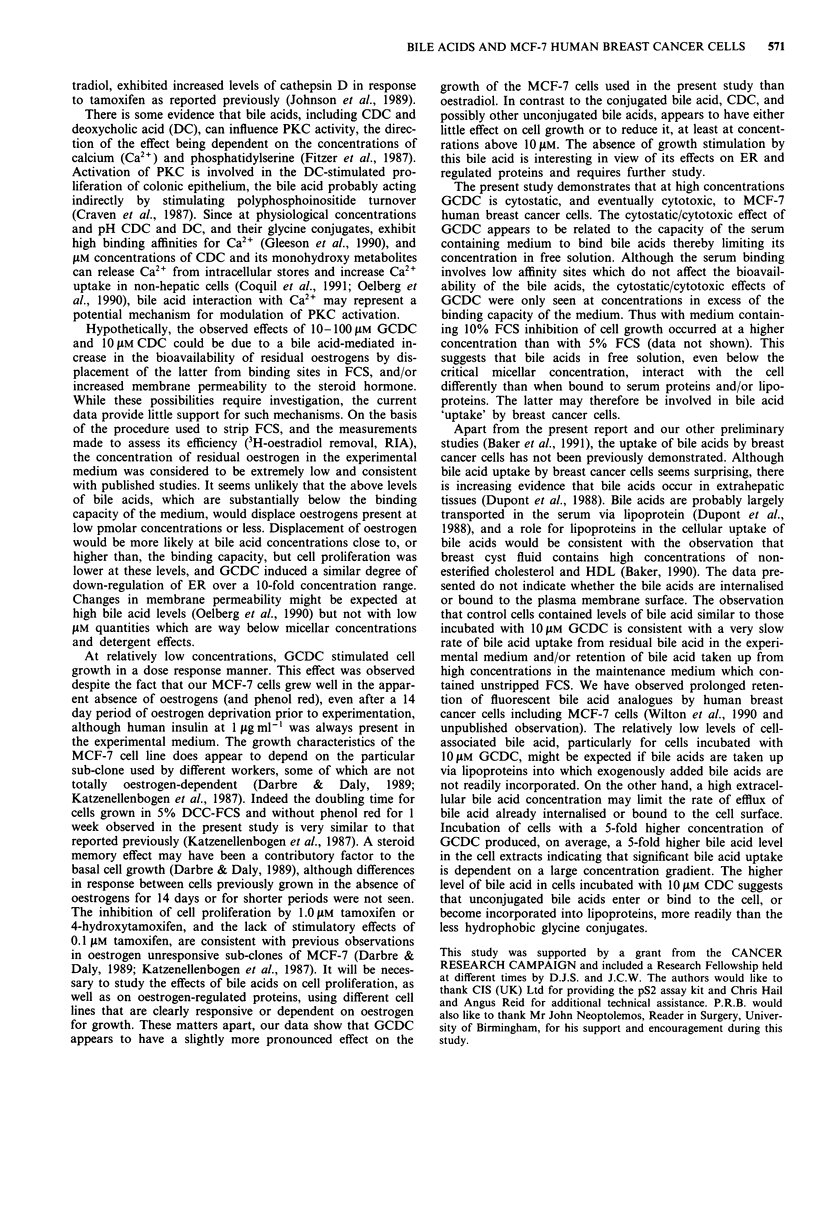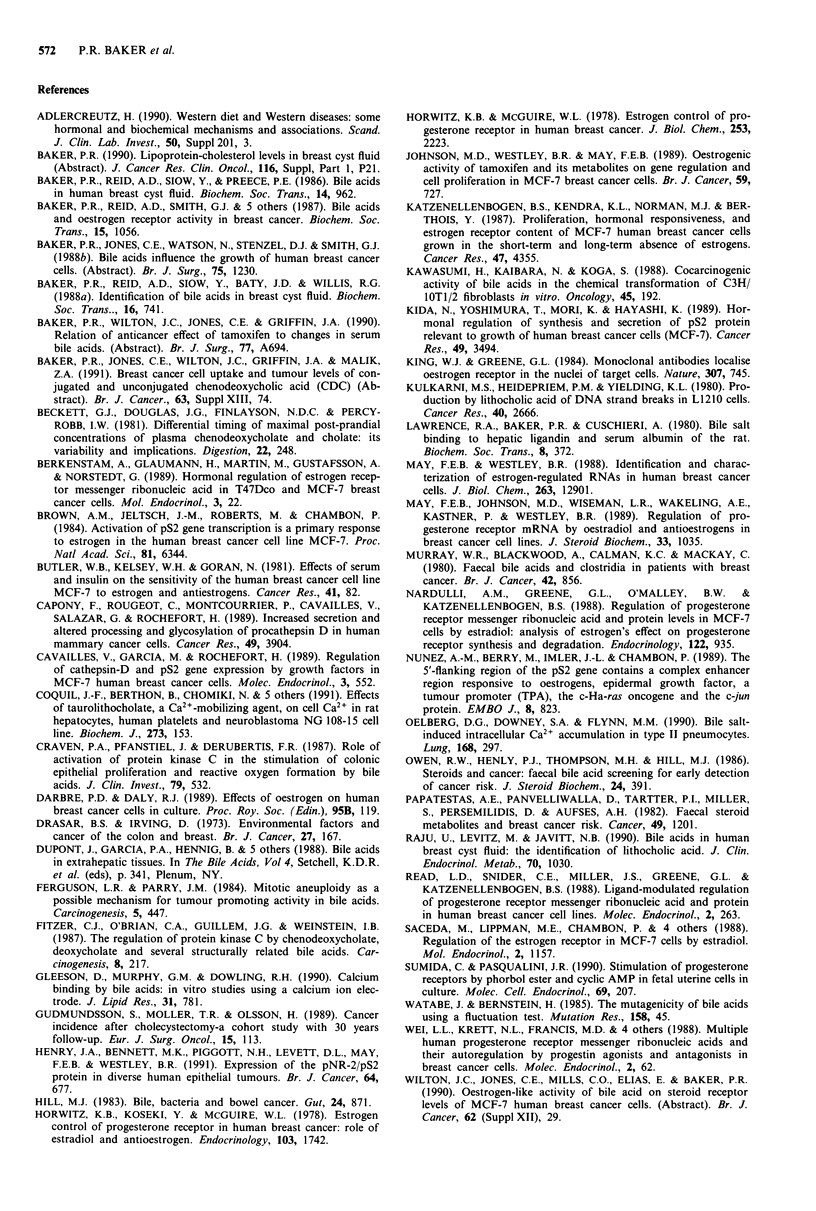# Bile acids influence the growth, oestrogen receptor and oestrogen-regulated proteins of MCF-7 human breast cancer cells.

**DOI:** 10.1038/bjc.1992.115

**Published:** 1992-04

**Authors:** P. R. Baker, J. C. Wilton, C. E. Jones, D. J. Stenzel, N. Watson, G. J. Smith

**Affiliations:** Department of Surgery, University of Birmingham, UK.

## Abstract

The effects of the major human serum bile acid, glycochenodeoxycholic acid (GCDC), as well as unconjugated chenodeoxycholic acid (CDC), on the MCF-7 human breast cancer cell line have been studied in vitro under oestrogen and bile acid deprived culture conditions. GCDC increased the growth of the breast cancer cells over the range 10-300 microM. At concentrations in excess of the bile acid binding capacity of the medium cell growth was prevented. In contrast 10 microM CDC tended to reduce cell growth. Oestrogen (ER) and progesterone (PgR) receptors, pS2 and total cathepsin D were quantified by monoclonal antibody based immunoassays. Ten to 100 microM GCDC and 10 microM CDC down-regulated ER protein and this was accompanied by induction of the oestrogen-regulated proteins PgR, pS2 and possibly cathepsin D, including increased secretion of the latter two proteins into the culture medium. All these changes were quantitatively similar to those observed with 10 nM oestradiol. The bile acid effects on ER and PgR were not due to interference with the assay procedures. Cells incubated with 50 microM GCDC or 10 microM CDC had higher pmolar concentrations of the bile acids than controls. This study suggests that naturally occurring bile acids influence the growth and steroid receptor function of human breast cancer cells.


Br. J. Cancer (1992), 65, 566–572 © Macmillan Press Ltd., 1992

### Bile acids influence the growth, oestrogen receptor and oestrogen-regulated proteins of MCF-7 human breast cancer cells

P.R. Baker[1], J.C. Wilton[1], C.E. Jones[1], D.J. Stenzel[2], N. Watson[2] & G.J. Smith[2]

[1]*Department of Surgery, University of Birmingham, Queen Elizabeth Hospital, Edgbaston, Birmingham B15 2TH;* [2]*Department of Surgery, University of Dundee, Ninewells Hospital, Dundee DD1 9SY, UK.*

**Summary** The effects of the major human serum bile acid, glycochenodeoxycholic acid (GCDC), as well as unconjugated chenodeoxycholic acid (CDC), on the MCF-7 human breast cancer cell line have been studied *in vitro* under oestrogen and bile acid deprived culture conditions. GCDC increased the growth of the breast cancer cells over the range 10–300 μM. At concentrations in excess of the bile acid binding capacity of the medium cell growth was prevented. In contrast 10 μM CDC tended to reduce cell growth. Oestrogen (ER) and progesterone (PgR) receptors, pS2 and total cathepsin D were quantified by monoclonal antibody based immunoassays. Ten to 100 μM GCDC and 10 μM CDC down-regulated ER protein and this was accompanied by induction of the oestrogen-regulated proteins PgR, pS2 and possibly cathepsin D, including increased secretion of the latter two proteins into the culture medium. All these changes were quantitatively similar to those observed with 10 nM oestradiol. The bile acid effects on ER and PgR were not due to interference with the assay procedures. Cells incubated with 50 μM GCDC or 10 μM CDC had higher pmolar concentrations of the bile acids than controls. This study suggests that naturally occurring bile acids influence the growth and steroid receptor function of human breast cancer cells.

Although bile acids have been considered to play a role in the aetiology and/or growth of colorectal cancer (Hill, 1983) little attention has been paid to the possible activity of these steroidal compounds in breast cancer. Epidemiological studies have demonstrated that rates of breast and colon cancers are highly correlated with each other and with high fat and animal protein diets (Drasar & Irving, 1973). In the few studies published the data suggest that women with breast cancer may have differences in the faecal excretion of bile acids compared with controls; decreased (Murray *et al.*, 1980) and increased (Papatestas *et al.*, 1982) faecal concentrations, and altered ratio of individual bile acids (Owen *et al.*, 1986) have been reported. Long-term follow-up of women undergoing cholecystectomy has revealed an increased risk of breast cancer which increased with time after operation (Gudmundsson *et al.*, 1989). In contrast, by reducing the enterohepatic circulation of oestrogens and bile acids, the high consumption of fibre and fermented milk products, particularly in combination with low fat and animal protein intake, may provide some protection against breast cancer (Adlercreutz, 1990).

These findings, while by no means conclusive, imply that breast cancer patients are subject to changes in circulating levels of bile acids and consequently the potential exposure of breast tissue and/or tumour. In support of the latter possibility is our observation, now confirmed by others, that breast cyst fluid contains very high levels of bile acids, notably the glycine conjugates of chenodeoxycholic (CDC), deoxycholic and cholic acids (Baker *et al.*, 1986 and 1988*a*; Raju *et al.*, 1990).

Bile acids have been shown to produce DNA changes in murine lymphoblastoma cells and in yeast cells (Ferguson & Parry, 1984; Kulkarni *et al.*, 1980). Several bile acids are mutagenic at non-toxic doses (Watabe & Bernstein, 1985), and have co-carcinogenic activity on a murine fibroblast cell line (Kawasumi *et al.*, 1988).

The action of bile acids on human breast cancer cells has not previously been studied apart from our own preliminary reports (Baker *et al.*, 1988*b*; Wilton *et al.*, 1990). The predominant serum bile acid in healthy women and women with breast cancer is glycochenodeoxycholic acid (GCDC) (Baker *et al.*, 1987). During tamoxifen treatment of elderly breast cancer patients the serum GCDC concentration is reduced while there is some increase in the unconjugated bile acid fraction containing CDC (Baker *et al.*, 1990). Perhaps complimentary to this latter observation are the lower levels of unconjugated bile acids (cholic, CDC & deoxycholic) in the serum of women with oestrogen receptor (ER) rich tumours and in healthy women taking oral contraceptive steroids continuously for over 6 years (Baker *et al.*, 1987). We have therefore investigated the effects of GCDC and CDC on the human breast cancer cell line MCF-7, with particular emphasis on changes in ER and the oestrogen-regulated proteins progesterone receptor (PgR), pS2 and cathepsin D (Brown *et al.*, 1984; Capony *et al.*, 1989; Horwitz *et al.*, 1978; Johnson *et al.*, 1989; Kida *et al.*, 1989; May *et al.*, 1989; May & Westley, 1988).

Correspondence: P.R. Baker, University Department of Surgery, Queen Elizabeth Hospital, Edgbaston, Birmingham B15 2TH, UK.
Received 9 September 1991; and in revised form 13 November 1991.

### Methods

### *Materials*

MCF-7 human breast cell lines were kindly provided by Dr R.E. Leake (Department of Biochemistry, University of Glasgow) and Dr A.E. Wakeling (ICI, Cheshire, England), and are identified as MCF-7(REL) and MCF-7(AEW) respectively. The sub-culture obtained from Dr Wakeling originated from cells supplied by the Glasgow laboratory. Culture media, supplements, NUNC plastic culture dishes and flasks, and mycoplasma free foetal calf serum (FCS) were obtained from Flow Laboratories (High Wycombe, Buckinghamshire, England), Gibco Ltd (Paisley, Scotland and Uxbridge, England), and Sigma Ltd (Poole, England). Human monocomponent insulin was from Novo Industri (Copenhagen, Denmark). Bile acids were of the highest available purity from Sigma and Calbiochem (Bishops Stortford, England). Tamoxifen, 4-hydroxytamoxifen and ICI 164384 were kindly donated by ICI. Radiolabelled compounds were from Amersham International, England, and all other reagents were analytical grade, or the highest grade available, from Sigma or BDH (Atherstone, England or Hayman Ltd (Witham, England)). All aqueous reagent solutions and culture media were prepared with 0.22 μm filtered ultra-pure water from a Milli-Q unit (Millipore, England) fed with water from a double-still or reverse osmosis unit.

*Dextran-coated charcoal treated FCS*

Oestrogens and bile acids were removed from FCS by two or three 1 h treatments at 56°C with dextran-coated charcoal (DCC) (Horwitz *et al.*, 1978). After the final centrifugation step the FCS was passed through 0.22 μm sterile cellulose acetate filters (Sartorius, England), which remove charcoal fines, and checked for sterility on nutrient broth. The level of residual $^{3}$H-oestradiol in DCC-treated FCS (DCC-FCS) was $<1-2\%$. Residual oestradiol in DCC-FCS was undetectable by RIA. The amount of residual $^{14}$C-glycocholic acid by the above procedure was 1–3%. Radioimmunoassay (RIA) of conjugated and unconjugated chenodeoxycholic acid (see below) demonstrated that untreated FCS contained a relatively high level of the bile acids (e.g. $>16$ nmol ml$^{-1}$) which was reduced to $<3\%$ in DCC-FCS. These observations indicate that the final concentration of the bile acids in media containing 5% DCC-FCS was about 25 nM which compares with the μM concentrations of exogenous bile acids used in this study. Approximately 80% of endogenous insulin in FCS was removed by the DCC procedure as assessed by RIA (Incstar Corp, Minnesota, USA), whereas about 60% of $^{14}$C-cholesterol remained in DCC-FCS.

*Bile acid binding capacity of FCS and DCC-FCS*

A volume of DCC-FCS containing 100 μg albumin was incubated with a saturating concentration (100 μM) of glycocholic acid (GC) or chenodeoxycholic acid (CDC) containing 100 nCi of $^{14}$C-labelled GC or CDC respectively in a total volume of 1.0 ml of 10 mM phosphate buffer pH 7.4 for 3 h at 37°C with repeated mixing. Three 40 μl aliquots of the incubate were taken for counting (total radioactivity) and 800 μl was passed through a YMT ultrafiltration membrane in a MPS-1 unit (Amicon, Stonehouse, England) by centrifugation at 1,000 *g* for 10 min. The resulting ultrafiltrate (3 × 40 μl) was added to 10 ml Scintran Cocktail T (BDH) and the radioactivity counted (free or non-protein bound activity). The amount of bound radioactivity was calculated and used as an expression of bile acid binding capacity.

*Cell culture procedures*

Cells were grown at 37°C with 5% $CO_2$ in air and were maintained prior to experimentation in DME:Ham's F-10 (1:1) or DME media, both with phenol red, and containing 10% or 5% FCS and supplemented with glutamine (2 mM), penicillin (50 IU ml$^{-1}$), streptomycin (50 μg ml$^{-1}$) and usually 1% non-essential amino acids (NEAA) and insulin (1 μg ml$^{-1}$). For experimentation cells growing in log-phase were transferred to phenol red free DME:Ham's F-12 (1:1) medium containing 5% DCC-FCS and the above supplements (experimental medium) in 24 well plates, 35 mm dishes or flasks (75 or 80 cm$^{2}$). In many cases cells were grown in this medium for 7–14 days prior to addition of test agents. For growth experiments cells were usually plated out in triplicate into 35 mm petri dishes, while for the study of receptors and proteins 75 or 80 cm$^{2}$ flasks were used in order to obtain sufficient numbers of cells. In these experiments triplicate cultures were not set up for practical reasons, but independent experiments were run on several separate occasions. Each experiment within a series was performed using newly cultured cells of the same passage number obtained from liquid nitrogen storage. Bile acids, oestradiol and antioestrogens were added to experimental medium in ethanol (0.1% final concentration or less); controls received the same amount of ethanol alone. These agents were added in fresh medium when cells were plated out or 24 h later, and then every 2–3 days when medium was changed. Cells were harvested with trypsin/EDTA (Flow Laboratories) and washed with medium and phosphate buffered saline (PBS) prior to counting or homogenisation.

*Cell counting*

Intact cells were repeatedly passed through a plastic pipette tip to obtain a single cell suspension and then counted on a Coulter counter or with a haemocytometer. In later studies cells were counted as nuclei using the method of Butler *et al.*, 1981.

*Cell homogenisation*

*'Cytosol' preparation* Harvested cells were washed several times in PBS, suspended in 10 mM Tris buffer pH 7.4 containing 1.0 mM EDTA, 15% glycerol and 0.25 mM dithiothreitol (DTT) and homogenised on ice with an Ultra-Turrax (model T 18/10, Sartorius, England) for 3 × 10 s at near maximum speed. The supernatant obtained after centrifugation at 600 *g* for 10 min and 5,000 *g* for 30 min at 4°C was used as a 'cytosol' preparation for immediate assay of steroid receptors. Cytosol prepared from this supernatant by centrifugation at 100,000 *g* in a Beckman model L2-65B ultracentrifuge was shown to give very similar levels of ER by EIA (see below) as the lower speed preparation (99 ± 15%, $n = 4$).

*'Whole cell' extraction* For the later studies in which ER and PgR were assayed in 'whole cell' extracts, harvested cells were washed several times in PBS, suspended in 10 mM HEPES buffer pH 7.4, containing 0.4 M KCl, 5 mM sodium molybdate, 1.5 mM EDTA, 10% glycerol and 0.5 mM DTT and homogenised as above. The supernatant after centrifugation at 2,000 *g* for 10 min and 5,000 *g* for 30 min at 4°C was used for immediate assay of ER and PgR. Aliquots of the extract as well as culture medium were stored at −40°C for other assays.

*Steroid receptor assays*

ER and PgR were determined in duplicate in 'cytosol' and 'whole cell' preparations with the enzyme immunoassays (EIA) supplied by Abbott Diagnostics (Maidenhead, England). Both the quality control (QC) material supplied with the kits and an external QC supplied by the EORTC were assayed with each batch of cell samples. Precision (CV%) of the assays was as follows: ER $<100$ fmol mg$^{-1}$, 4.8% ($n = 27$), ER $>100$ fmol mg$^{-1}$, 5.0% ($n = 64$), PgR $<100$ fmol mg$^{-1}$, 6.0% ($n = 23$), PgR = 100–500 fmol mg$^{-1}$, 2.3% ($n = 32$), PgR $>500$ fmol mg$^{-1}$, 8.6% ($n = 30$).

*pS2, Cathepsin D and GCDC assays*

The oestrogen-regulated proteins pS2 and cathepsin D (52 kd pro-cathepsin D plus the active enzyme) were determined with the two site immunoradiometric assays (ELSA-PS2 and ELSA-CATH-D) supplied by CIS (UK) Ltd. (High Wycombe, England). Assays were performed in duplicate on stored 'whole cell' extracts or culture medium diluted appropriately with the diluent provided in the kits. The high KCl concentration of the homogenisation buffer had no effect on the quantification of either protein (personal observation and information from CIS). The CV for the pS2 assay was 5.4% ($n = 50$) over the range 29–2,006 pg ml$^{-1}$ diluted sample. For the cathepsin D assay the CV was 5.4% ($n = 50$) for values $<1,000$ fmol ml$^{-1}$ diluted sample and 10.7% ($n = 50$) for values $>1,000$ fmol ml$^{-1}$ diluted sample.

GCDC and CDC in 'whole cell' extracts were quantified with the same radioimmunoassay supplied by Farmos Diagnostica (Pharmacia Ltd, Milton Keynes, England) using the kit procedure for serum which involves extraction into 95% ethanol prior to assay. The CV was 8.6% ($n = 29$). This assay was also performed on whole cells by dissolving the washed (3 ×) intact cell pellet in 1 ml 5% NaOH at 80°C for 1.5 h, diluting with 9 ml of water and extracting the bile acids into methanol on a 100 mg Bondelut C18 solid phase cartridge (Jones Chromatography, Hengoed, Wales). The dried methanol extract was then extracted into 95% ethanol ready for assay.

*Protein assay*

Protein levels in cell preparations were determined using the Bio-Rad protein assay with bovine serum albumin as standard.

*Statistical analysis*

Precision of an assay is expressed as the coefficient of variation (CV%) obtained from series of $n$ duplicates. Results are usually expressed as mean ± s.d. Statistical analysis was performed by analysis of variance (AOV) with the Dunnett test for multiple comparisons with control, or by paired $t$-test; the 5% level was considered to indicate a signficant difference.

### Results

*Bile acid binding capacity of DCC-FCS*

The bile acid binding capacity of DCC-FCS in $\mu$mol ml$^{-1}$ undiluted serum was 2.4 ± 0.4 ($n$ = 2) and 11.2 ± 0.3 ($n$ = 4) for GC and CDC respectively. From these values, the concentration of protein-bound bile acid in culture medium containing 5% DCC-FCS would be 118 ± 20 $\mu$M for GC and 558 ± 16 $\mu$M for CDC. Since bile acid binding to serum albumin is a characteristic of the degree of hydroxylation of the steroid nucleus rather than glycine conjugation (Lawrence *et al.*, 1980), these data suggest that medium containing 5% DCC-FCS would have a GCDC protein binding capacity of approximately 500 $\mu$M.

*Cell growth*

Early studies were performed using MCF-7(REL) cells. The effect of increasing concentration of GCDC on cell growth is illustrated in Figure 1. Growth stimulation in this and other similar experiments was clearly seen at concentrations of GCDC between 10 and 300 $\mu$M, although above 100 $\mu$M growth stimulation was not maintained after 8 days. Above 500 $\mu$M GCDC was cytostatic, possibly because the protein binding capacity of the medium was exceeded. At high concentrations (1 mM) GCDC was cytotoxic (data not shown). Optimum growth stimulation appeared to occur at 100 $\mu$M GCDC and this produced significantly increased cell numbers after 7 to 10 days of culture (25.0 ± 8.7 $10^4$ cells cm$^{-2}$; control = 11.9 ± 4.0, $P < 0.005$, $n = 7$), and a significant decrease in doubling time (1.8 ± 0.4 days; control = 2.6 ± 0.4, $P < 0.02$, $n = 4$). This effect did not seem to depend on 'conditioning' of the cells in oestrogen and bile acid depleted medium for 10 days prior to addition of GCDC. Preliminary studies with a series of glycine conjugated and unconjugated bile acids at 100 $\mu$M suggests that at this concentration, glycoursodeoxycholic, ursodeoxycholic and cholic acids have little effect on cell growth, while glycolithocholic, lithocholic, deoxycholic and CDC are cytotoxic, and glycine conjugates of cholic, deoxycholic and CDC all stimulate cell growth.

**Figure 1** Effect of different concentrations of GCDC on growth of MCF-7(REL) cells. Cells were grown for 10 days in experimental medium and then 6 × $10^4$ cells were plated out into 35 mm dishes in 2 ml of fresh medium without (0) and with the $\mu$M concentrations of GCDC shown in the figure. Medium was changed every 2 days and intact cells were counted as described under Methods. Doubling times (days) for log-phase of growth for the GCDC concentration shown in parentheses were; 2.50 (0), 2.42 (0.5), 2.11 (1.0), 1.72 (10), 1.66 (50), 1.40 (100), 1.32 (200), 1.41 (300), 1.83 (500).

Pre-conditioning cells in oestrogen and bile acid depleted medium prior to experimentation was routinely performed in subsequent studies with MCF-7 (AEW) cells. Although this was done most commonly for 14 days the effects on growth and steroid receptors seen with shorter times (2 or 7 days) were very similar and therefore results have been combined. Lower concentrations of bile acids were used in these experiments in an attempt to study more physiological levels. In these experiments ($n$ = 4–5) a reduction in mean doubling time was seen for 10 nM oestradiol (1.8 ± 0.4 days), 10 $\mu$M GCDC (1.6 ± 0.2) and 50 $\mu$M GCDC (1.5 ± 0.1) compared with control (1.9 ± 0.2). These differences were not significant on multiple comparisons analysis (Dunnett test), although the effect of 50 $\mu$M GCDC was significant ($P < 0.02$) when compared with controls by paired $t$-test. In three of four experiments 10 $\mu$M CDC reduced cell growth giving a doubling time of 2.2 ± 0.5 days. The MCF-7(AEW) cells used in this study grew well in the oestrogen and bile acid depleted medium containing 1 $\mu$g ml$^{-1}$ human insulin, and the growth stimulation by 10 nM oestradiol was modest. In a separate series of experiments cell doubling times were greatly reduced from control (3.0 ± 0.2 days; $n = 5$) by 1 $\mu$M tamoxifen (5.8 ± 0.2) and 4-hydroxytamoxifen (6.1 ± 0.3), while tamoxifen at 0.1 $\mu$M had little or no effect on cell proliferation.

*Steroid receptors, pS2 and cathepsin D*

Since concentrations of GCDC > 100 $\mu$M eventually resulted in reduced cell growth (Figure 1) the effects of the bile acid on these proteins were studied at 10–100 $\mu$M. Cells were harvested after 7 days culture without or with 10 nM oestradiol or bile acids. ER levels in 'cytosol' of MCF-7(REL) cells were reduced by 10 nM oestradiol and 100 $\mu$M GCDC as shown in Table I. In later experiments MCF-7(AEW) cells were grown in oestrogen and bile acid depleted medium for 2 to 14 days prior to addition of lower, and more physiological, levels of GCDC and CDC, and a similar degree of down-regulation of ER in 'whole cell' extracts was observed (Figure 2).

The down-regulation of ER by bile acids and oestradiol was accompanied by significant induction of PgR and pS2 by these agents in the same series of experiments (Figures 3 and 4). Furthermore, the substantial secretion of the latter protein into the culture medium observed in controls was greatly increased by both bile acids (Table II). Although there appeared to be some increase in the mean levels of total cathepsin D (pmol mg$^{-1}$) in these 'whole cell' extracts in response to oestradiol (28 ± 10, $n = 5$), 10 $\mu$M GCDC (29 ± 14, $n = 4$), 50 $\mu$M GCDC (28 ± 19, $n = 6$) and CDC (30 ± 9, $n = 4$), comparisons with controls (22 ± 13, $n = 6$) did not reach significance. However, in the two experiments in which cathepsin D was assayed in the medium 10 and 50 $\mu$M GCDC appeared to approximately double the secretion of the protein (Table II).

Tamoxifen and 4-hydroxytamoxifen at 1 $\mu$M increased ER in 'whole cell' extracts relative to oestradiol stimulation (327 ± 65%, $n = 3$; and 403 ± 57%, $n = 2$, respectively) and decreased PgR (15 ± 3% and 13 ± 3%, respectively). On the other hand, 0.1 $\mu$M tamoxifen acted as an oestrogen, down-regulating ER (58 ± 22% of control, $n = 5$) and inducing PgR (344 ± 202% of control, $n = 5$) despite having little effect on cell growth. ICI 164384 (0.1 $\mu$M) reduced the levels of both steroid receptors relative to oestradiol stimulation (23 ± 5% and 1.3 ± 0.7%, $n = 2$, for ER and PgR respectively) and this effect was partially reversed by 10 nM oestradiol. Tamoxifen at 0.1 $\mu$M increased cellular cathepsin D levels (40 ± 25 pmol mg$^{-1}$, $n = 5$) compared with control

**Table I** Effect of oestradiol and 100 μM GCDC on 'cytosol' ER levels of MCF-7(REL) cells

| | *fmol mg⁻¹ protein* | | | *% of Control* | |
|---|---|---|---|---|---|
| *Experiment* | *Control* | *E2* | *GCDC* | *E2* | *GCDC* |
| 1 | 65 | 30 | 49 | 46 | 75 |
| 2 | 875 | 186 | 369 | 21 | 42 |
| 3 | 224 | 17 | 48 | 8 | 21 |
| mean ± s.d. | | | | 25 ± 19 | 46 ± 27 |

| | *fmol 10⁻⁶ cells* | | | *% of Control* | |
|---|---|---|---|---|---|
| *Experiment* | *Control* | *E2* | *GCDC* | *E2* | *GCDC* |
| 1 | 10 | 7 | 7 | 70 | 70 |
| 2 | 97 | 39 | 36 | 40 | 37 |
| 3 | 58 | 3 | 10 | 5 | 17 |
| mean ± s.d. | | | | 38 ± 33 | 41 ± 27 |

$10^6$ cells were plated out and grown in 20 ml experimental medium in 80 $cm^2$ flasks for 7 days (late log-phase) without (Control) and with 10 nM oestradiol (E2) or 100 μM GCDC. Medium was changed every 2–3 days. The results are for three independent experiments with newly cultured cells.

**Figure 2** ER levels (fmol $mg^{-1}$ protein) in MCF-7(AEW) cells grown in the presence of oestradiol, GCDC or CDC. Cells were grown for 2 (one experiment), 7 (one experiment) or 14 (four experiments) days in experimental medium before transfer to 75 $cm^2$ flasks containing 15 ml of medium. After 24 h the medium was replaced with fresh medium without (CTRL = Control) or with 10 nM oestradiol (E2), 10μM GCDC (GCDC10), 50μM GCDC (GCDC50) or 10μM CDC (CDC10). Medium was changed every 2–3 days. After 7 days (late log-phase) cells were harvested, homogenised to prepare a 'whole cell' extract and assayed as described under Methods. Results are expressed as mean (columns) and s.d. (error bars) for the number of experiments shown at the foot of each column. The *P* values above the columns are for analysis using the Dunnett test for multiple comparisons with CTRL (OAV gave $P = 0.011$).

**Figure 3** PgR levels (fmol $mg^{-1}$ protein) in MCF-7(AEW) cells grown in the presence of oestradiol, GCDC or CDC. For details see legend to Figure 2 and Methods. (AOV gave $P = 0.0003$).

**Figure 4** pS2 levels (ng $mg^{-1}$ protein) in MCF-7(AEW) cells grown in the presence of oestradiol, GCDC or CDC. For details see legend to Figure 2 and Methods. (AOV gave $P =$ 0.0018).

(25 ± 13). At 1 μM tamoxifen also increased cellular cathepsin D relative to control (204 ± 97%, $n = 2$) as well as the amount secreted into the medium (253 ± 16%, $n = 2$). Interestingly, 0.1 μM ICI 164384 increased cellular levels of cathepsin D in two of three experiments but had little effect on secretion of the proteinase into the culture medium. Very low levels of secreted pS2 were obtained when cells were grown for 6 days in medium without FCS (18 ± 9% of control, $n = 2$). Antioestrogen effects on pS2 were not studied.

### *Effect of GCDC on assay of ER and PgR*

'Whole cell' extracts of MCF-7(AEW) cells grown without and with 10 nM oestradiol were mixed with varying concentrations of GCDC (0, 1, 5, 10, 50 and 100 μM final concentration in cytosol) prior to assay of ER and PgR. Steroid receptor values were generally unaffected by GCDC apart from the high PgR levels induced by oestradiol where a 20% decrease was seen at 50 and 100 μM GCDC. These concentrations are far in excess of the levels of bile acids detected in cell extracts (see below). Over the concentration range 5–100 μM GCDC the recovery of the added bile acid from the cytosol was 89 ± 8%.

### *GCDC and CDC in 'whole cell' extracts and whole cells*

The 'whole cell' extracts in which oestrogen receptor and regulated proteins were measured were assayed for GCDC/CDC content by RIA. Control cells contained low levels of the bile acids (2.5 ± 2.9 pmol $10^{-6}$ cells) consistent with the residual concentration of bile acids in the medium (24 nM).

**Table II** Secretion of pS2 and cathepsin D by MCF-7(AEW) cells into the culture medium in response to oestradiol, GCDC and CDC

| | *Secreted pS2 (ng $10^{-6}$ cells $24\,h^{-1}$)* | | | | |
|---|---|---|---|---|---|
| *Experiment* | *Control* | *E2* | *GCDC10* | *GCDC50* | *CDC10* |
| 1 | 46.3 | 150.9[a] | 342.3 | 345.5 | 213.8 |
| 2 | 10.0 | – | 92.3 | 120.7 | – |
| 3 | 30.6 | – | – | 107.2 | 54.2 |
| 4 | 60.2 | – | – | 233.4 | 110.3 |
| mean ± s.d. | 36.8 ± 21.6 | | | 200.7 ± 109.6 | 126.1 ± 81.0 |

| | *Secreted cathepsin D (pmol $10^{-6}$ cells $72\,h^{-1}$)* | | | | |
|---|---|---|---|---|---|
| *Experiment* | *Control* | *E2* | *GCDC10* | *GCDC50* | *CDC10* |
| 1 | 1.25 | 1.68[a] | 3.13 | 2.56 | 1.71 |
| 2 | 1.54 | – | 2.59 | 2.53 | – |

For experiments 1 and 2 (both proteins) cells were grown as described in the legend to Figure 2 (7 or 14-day pre-conditioning) and 72 h medium was collected after 7 days of culture when the cells were harvested. For experiments 3 and 4 (pS2 only) $1-3 \times 10^5$ cells, pre-conditioned for 7 days, were plated out and grown in 1 ml of experimental medium in 35 mm dishes and 48 h medium after 6 days culture was assayed for pS2. ([a]The effect of E2 on pS2 and cathepsin D secretion was only studied in one experiment).

While extracts of cells incubated with 10 µM GCDC had similar levels (3.8 ± 3.0), cells incubated with 50 µM GCDC (15.3 ± 7.6, $P<0.01$, Dunnett test) or 10 µM CDC (11.4 ± 3.6, $P<0.05$) contained significantly higher amounts of bile acid. These latter results have been confirmed in separate experiments in which whole cells, harvested every 2 days and processed as described above, had increasingly higher levels of GCDC or CDC compared with controls; this indicates that the cell-associated bile acid is not due to simple cell adherence or residual medium. At 8 days the bile acid levels of cells incubated with 10 µM CDC and 50 µM GCDC were 17.0 ± 2.2 and 34.2 ± 1.4 pmol $10^{-6}$ cells ($n$ = 2) respectively compared with 1.8 ± 0.6 in controls.

### Discussion

The most significant aspect of the present study is the demonstration that GCDC and CDC appear to exert an oestrogen-like effect on MCF-7 cells by down-regulating ER levels and inducing the oestrogen regulated proteins PgR and pS2, and possibly cathepsin D. The down-regulation of ER was observed for both 'cytosol' and 'whole cell' levels of the receptor which is localised to the nucleus *in vivo* (King & Greene, 1984) from where it can be extracted into the soluble supernatant by homogenisation with buffer containing 0.4 M KCl (Saceda *et al.*, 1988). Nevertheless, the decrease in 'cytosolic' ER by 100 µM GCDC in early experiments was similar to that seen in 'whole cell' extracts of cells exposed to 10–50 µM GCDC and 10 µM CDC. The lower levels of bile acids are within the range of post-prandial serum concentrations (Beckett *et al.*, 1981). The use of a monoclonal antibody enzyme immunoassay (King & Greene, 1984) provided a very sensitive and precise method of measuring receptor protein, and the levels of ER in control cells and cells treated with oestradiol observed here were very similar to those in a previous study using the same assay kit (Saceda *et al.*, 1988). Furthermore, the decrease in ER protein after 7 days oestradiol treatment agrees well with the down-regulation of ER mRNA after 2 days reported by others (Berkenstam *et al.*, 1989; Saceda *et al.*, 1988). The present study shows that the degree of down-regulation of ER by 10–100 µM GCDC and 10 µM CDC was very similar to that seen with 10 nM oestradiol.

The accompanying induction of PgR, pS2 and possibly also cathepsin D by the two bile acids was also similar to that seen with 10 nM oestradiol. The degree of oestradiol induction of PgR mRNA and protein is reported to be 2–10- and 3–20-fold respectively (Horwitz & McGuire, 1978; Katzenellenbogen *et al.*, 1987; Nardulli *et al.*, 1988; Wei *et al.*, 1988), which is consistent with our data for oestradiol and bile acids, although larger increases have been observed (May *et al.*, 1989; Read *et al.*, 1988). The induction of PgR by oestrogen appears to be due to changes at the transcriptional, or possibly post-transcriptional, level, rather than alteration of protein degradation (Read *et al.*, 1988; Nardulli *et al.*, 1988; Wei *et al.*, 1988), but whether this applies to bile acids cannot be concluded from the present study. The effect of progestins on bile acid-induced PgR levels might provide a useful insight into this mechanism (Wei *et al.*, 1988). ER and PgR response to 0.1, 1 µM tamoxifen (oestrogenic), 1 µM tamoxifen or 4-hydroxytamoxifen (antioestrogenic) and ICI 164384 are consistent with previous reports of oestrogen-responsive MCF-7 cells (Horwitz *et al.*, 1978; Johnson *et al.*, 1989; May *et al.*, 1989; Read *et al.*, 1988).

The secreted protein pS2/pNR2 appears to be under primary oestrogen control in breast tumours and cell lines, but is also expressed by several epithelial tumours as well as some normal tissues (Brown *et al.*, 1984; Cavailles *et al.*, 1989; Henry *et al.*, 1991; May & Westley, 1988). In addition pS2, via activation of its enhancer element, is also induced by insulin, IGF-I, bFGF, EGF, increased intracellular cAMP, stimulation of protein kinase C (PKC) by phorbol esters and some oncogene products (Cavailles *et al.*, 1989; Nunez *et al.*, 1989). Many of these factors are known to mediate the mitogenic activity of oestrogens, but the pS2 protein itself is not a mitogen and its function is still unknown (Kida *et al.*, 1989). As well as inducing pS2, activation of PKC can result in increased PgR levels (Sumida & Pasqualini, 1990). The induction of pS2 and PgR by the bile acids was of the same order as with the above agents, and it is possible that GCDC and CDC act via similar mechanisms.

While the bile acids increased the secretion of cathepsin D into the culture medium, the changes in cellular levels were modest, as indeed was the case with oestradiol. Taken together as a measure of the total increase in cathepsin D, our, albeit limited data, suggest that some induction of cathepsin D by bile acids occurred, but further studies are required to confirm this including the use of cells in which oestradiol significantly induces the protein. Cathepsin D is expressed constitutively by ER+ and ER– breast cancer cells although it is regulated by oestrogens in the former (Capony *et al.*, 1989; May & Westley, 1988). It is induced by many of the same factors that stimulate pS2 but not increased cAMP or PKC activation (Cavailles *et al.*, 1989). The MCF-7 cells used here, although responding poorly to oestradiol, exhibited increased levels of cathepsin D in response to tamoxifen as reported previously (Johnson *et al.*, 1989).

There is some evidence that bile acids, including CDC and deoxycholic acid (DC), can influence PKC activity, the direction of the effect being dependent on the concentrations of calcium ($Ca^{2+}$) and phosphatidylserine (Fitzer *et al.*, 1987). Activation of PKC is involved in the DC-stimulated proliferation of colonic epithelium, the bile acid probably acting indirectly by stimulating polyphosphoinositide turnover (Craven *et al.*, 1987). Since at physiological concentrations and pH CDC and DC, and their glycine conjugates, exhibit high binding affinities for $Ca^{2+}$ (Gleeson *et al.*, 1990), and μM concentrations of CDC and its monohydroxy metabolites can release $Ca^{2+}$ from intracellular stores and increase $Ca^{2+}$ uptake in non-hepatic cells (Coquil *et al.*, 1991; Oelberg *et al.*, 1990), bile acid interaction with $Ca^{2+}$ may represent a potential mechanism for modulation of PKC activation.

Hypothetically, the observed effects of 10–100 μM GCDC and 10 μM CDC could be due to a bile acid-mediated increase in the bioavailability of residual oestrogens by displacement of the latter from binding sites in FCS, and/or increased membrane permeability to the steroid hormone. While these possibilities require investigation, the current data provide little support for such mechanisms. On the basis of the procedure used to strip FCS, and the measurements made to assess its efficiency ($^3$H-oestradiol removal, RIA), the concentration of residual oestrogen in the experimental medium was considered to be extremely low and consistent with published studies. It seems unlikely that the above levels of bile acids, which are substantially below the binding capacity of the medium, would displace oestrogens present at low pmolar concentrations or less. Displacement of oestrogen would be more likely at bile acid concentrations close to, or higher than, the binding capacity, but cell proliferation was lower at these levels, and GCDC induced a similar degree of down-regulation of ER over a 10-fold concentration range. Changes in membrane permeability might be expected at high bile acid levels (Oelberg *et al.*, 1990) but not with low μM quantities which are way below micellar concentrations and detergent effects.

At relatively low concentrations, GCDC stimulated cell growth in a dose response manner. This effect was observed despite the fact that our MCF-7 cells grew well in the apparent absence of oestrogens (and phenol red), even after a 14 day period of oestrogen deprivation prior to experimentation, although human insulin at 1 $\mu g\,ml^{-1}$ was always present in the experimental medium. The growth characteristics of the MCF-7 cell line does appear to depend on the particular sub-clone used by different workers, some of which are not totally oestrogen-dependent (Darbre & Daly, 1989; Katzenellenbogen *et al.*, 1987). Indeed the doubling time for cells grown in 5% DCC-FCS and without phenol red for 1 week observed in the present study is very similar to that reported previously (Katzenellenbogen *et al.*, 1987). A steroid memory effect may have been a contributory factor to the basal cell growth (Darbre & Daly, 1989), although differences in response between cells previously grown in the absence of oestrogens for 14 days or for shorter periods were not seen. The inhibition of cell proliferation by 1.0 μM tamoxifen or 4-hydroxytamoxifen, and the lack of stimulatory effects of 0.1 μM tamoxifen, are consistent with previous observations in oestrogen unresponsive sub-clones of MCF-7 (Darbre & Daly, 1989; Katzenellenbogen *et al.*, 1987). It will be necessary to study the effects of bile acids on cell proliferation, as well as on oestrogen-regulated proteins, using different cell lines that are clearly responsive or dependent on oestrogen for growth. These matters apart, our data show that GCDC appears to have a slightly more pronounced effect on the growth of the MCF-7 cells used in the present study than oestradiol. In contrast to the conjugated bile acid, CDC, and possibly other unconjugated bile acids, appears to have either little effect on cell growth or to reduce it, at least at concentrations above 10 μM. The absence of growth stimulation by this bile acid is interesting in view of its effects on ER and regulated proteins and requires further study.

The present study demonstrates that at high concentrations GCDC is cytostatic, and eventually cytotoxic, to MCF-7 human breast cancer cells. The cytostatic/cytotoxic effect of GCDC appears to be related to the capacity of the serum containing medium to bind bile acids thereby limiting its concentration in free solution. Although the serum binding involves low affinity sites which do not affect the bioavailability of the bile acids, the cytostatic/cytotoxic effects of GCDC were only seen at concentrations in excess of the binding capacity of the medium. Thus with medium containing 10% FCS inhibition of cell growth occurred at a higher concentration than with 5% FCS (data not shown). This suggests that bile acids in free solution, even below the critical micellar concentration, interact with the cell differently than when bound to serum proteins and/or lipoproteins. The latter may therefore be involved in bile acid 'uptake' by breast cancer cells.

Apart from the present report and our other preliminary studies (Baker *et al.*, 1991), the uptake of bile acids by breast cancer cells has not been previously demonstrated. Although bile acid uptake by breast cancer cells seems surprising, there is increasing evidence that bile acids occur in extrahepatic tissues (Dupont *et al.*, 1988). Bile acids are probably largely transported in the serum via lipoprotein (Dupont *et al.*, 1988), and a role for lipoproteins in the cellular uptake of bile acids would be consistent with the observation that breast cyst fluid contains high concentrations of non-esterified cholesterol and HDL (Baker, 1990). The data presented do not indicate whether the bile acids are internalised or bound to the plasma membrane surface. The observation that control cells contained levels of bile acid similar to those incubated with 10 μM GCDC is consistent with a very slow rate of bile acid uptake from residual bile acid in the experimental medium and/or retention of bile acid taken up from high concentrations in the maintenance medium which contained unstripped FCS. We have observed prolonged retention of fluorescent bile acid analogues by human breast cancer cells including MCF-7 cells (Wilton *et al.*, 1990 and unpublished observation). The relatively low levels of cell-associated bile acid, particularly for cells incubated with 10 μM GCDC, might be expected if bile acids are taken up via lipoproteins into which exogenously added bile acids are not readily incorporated. On the other hand, a high extracellular bile acid concentration may limit the rate of efflux of bile acid already internalised or bound to the cell surface. Incubation of cells with a 5-fold higher concentration of GCDC produced, on average, a 5-fold higher bile acid level in the cell extracts indicating that significant bile acid uptake is dependent on a large concentration gradient. The higher level of bile acid in cells incubated with 10 μM CDC suggests that unconjugated bile acids enter or bind to the cell, or become incorporated into lipoproteins, more readily than the less hydrophobic glycine conjugates.

This study was supported by a grant from the CANCER RESEARCH CAMPAIGN and included a Research Fellowship held at different times by D.J.S. and J.C.W. The authors would like to thank CIS (UK) Ltd for providing the pS2 assay kit and Chris Hail and Angus Reid for additional technical assistance. P.R.B. would also like to thank Mr John Neoptolemos, Reader in Surgery, University of Birmingham, for his support and encouragement during this study.

### References

ADLERCREUTZ, H. (1990). Western diet and Western diseases: some hormonal and biochemical mechanisms and associations. *Scand. J. Clin. Lab. Invest.*, **50**, Suppl 201, 3.

BAKER, P.R. (1990). Lipoprotein-cholesterol levels in breast cyst fluid (Abstract). *J. Cancer Res. Clin. Oncol.*, **116**, Suppl, Part 1, P21.

BAKER, P.R., REID, A.D., SIOW, Y., & PREECE, P.E. (1986). Bile acids in human breast cyst fluid. *Biochem. Soc. Trans.*, **14**, 962.

BAKER, P.R., REID, A.D., SMITH, G.J. & 5 others (1987). Bile acids and oestrogen receptor activity in breast cancer. *Biochem. Soc. Trans.*, **15**, 1056.

BAKER, P.R., JONES, C.E., WATSON, N., STENZEL, D.J. & SMITH, G.J. (1988*b*). Bile acids influence the growth of human breast cancer cells. (Abstract). *Br. J. Surg.*, **75**, 1230.

BAKER, P.R., REID, A.D., SIOW, Y., BATY, J.D. & WILLIS, R.G. (1988*a*). Identification of bile acids in breast cyst fluid. *Biochem. Soc. Trans.*., **16**, 741.

BAKER, P.R., WILTON, J.C., JONES, C.E. & GRIFFIN, J.A. (1990). Relation of anticancer effect of tamoxifen to changes in serum bile acids. (Abstract). *Br. J. Surg.*, **77**, A694.

BAKER, P.R., JONES, C.E., WILTON, J.C., GRIFFIN, J.A. & MALIK, Z.A. (1991). Breast cancer cell uptake and tumour levels of conjugated and unconjugated chenodeoxycholic acid (CDC) (Abstract). *Br. J. Cancer.*, **63**, Suppl XIII, 74.

BECKETT, G.J., DOUGLAS, J.G., FINLAYSON, N.D.C. & PERCY-ROBB, I.W. (1981). Differential timing of maximal post-prandial concentrations of plasma chenodeoxycholate and cholate: its variability and implications. *Digestion*, **22**, 248.

BERKENSTAM, A., GLAUMANN, H., MARTIN, M., GUSTAFSSON, A. & NORSTEDT, G. (1989). Hormonal regulation of estrogen receptor messenger ribonucleic acid in T47Dco and MCF-7 breast cancer cells. *Mol. Endocrinol.*, **3**, 22.

BROWN, A.M., JELTSCH, J.-M., ROBERTS, M. & CHAMBON, P. (1984). Activation of pS2 gene transcription is a primary response to estrogen in the human breast cancer cell line MCF-7. *Proc. Natl Acad. Sci.*, **81**, 6344.

BUTLER, W.B., KELSEY, W.H. & GORAN, N. (1981). Effects of serum and insulin on the sensitivity of the human breast cancer cell line MCF-7 to estrogen and antiestrogens. *Cancer Res.*, **41**, 82.

CAPONY, F., ROUGEOT, C., MONTCOURRIER, P., CAVAILLES, V., SALAZAR, G. & ROCHEFORT, H. (1989). Increased secretion and altered processing and glycosylation of procathepsin D in human mammary cancer cells. *Cancer Res.*, **49**, 3904.

CAVAILLES, V., GARCIA, M. & ROCHEFORT, H. (1989). Regulation of cathepsin-D and pS2 gene expression by growth factors in MCF-7 human breast cancer cells. *Molec. Endocrinol.*, **3**, 552.

COQUIL, J.-F., BERTHON, B., CHOMIKI, N. & 5 others (1991). Effects of taurolithocholate, a $Ca^{2+}$-mobilizing agent, on cell $Ca^{2+}$ in rat hepatocytes, human platelets and neuroblastoma NG 108-15 cell line. *Biochem. J.*, **273**, 153.

CRAVEN, P.A., PFANSTIEL, J. & DERUBERTIS, F.R. (1987). Role of activation of protein kinase C in the stimulation of colonic epithelial proliferation and reactive oxygen formation by bile acids. *J. Clin. Invest.*, **79**, 532.

DARBRE, P.D. & DALY, R.J. (1989). Effects of oestrogen on human breast cancer cells in culture. *Proc. Roy. Soc. (Edin.)*, **95B**, 119.

DRASAR, B.S. & IRVING, D. (1973). Environmental factors and cancer of the colon and breast. *Br. J. Cancer*, **27**, 167.

DUPONT, J., GARCIA, P.A., HENNIG, B. & 5 others (1988). Bile acids in extrahepatic tissues. In *The Bile Acids, Vol 4*, Setchell, K.D.R. *et al.* (eds), p. 341, Plenum, NY.

FERGUSON, L.R. & PARRY, J.M. (1984). Mitotic aneuploidy as a possible mechanism for tumour promoting activity in bile acids. *Carcinogenesis*, **5**, 447.

FITZER, C.J., O'BRIAN, C.A., GUILLEM, J.G. & WEINSTEIN, I.B. (1987). The regulation of protein kinase C by chenodeoxycholate, deoxycholate and several structurally related bile acids. *Carcinogenesis*, **8**, 217.

GLEESON, D., MURPHY, G.M. & DOWLING, R.H. (1990). Calcium binding by bile acids: in vitro studies using a calcium ion electrode. *J. Lipid Res.*, **31**, 781.

GUDMUNDSSON, S., MOLLER, T.R. & OLSSON, H. (1989). Cancer incidence after cholecystectomy-a cohort study with 30 years follow-up. *Eur. J. Surg. Oncol.*, **15**, 113.

HENRY, J.A., BENNETT, M.K., PIGGOTT, N.H., LEVETT, D.L., MAY, F.E.B. & WESTLEY, B.R. (1991). Expression of the pNR-2/pS2 protein in diverse human epithelial tumours. *Br. J. Cancer*, **64**, 677.

HILL, M.J. (1983). Bile, bacteria and bowel cancer. *Gut*, **24**, 871.

HORWITZ, K.B., KOSEKI, Y. & MCGUIRE, W.L. (1978). Estrogen control of progesterone receptor in human breast cancer: role of estradiol and antioestrogen. *Endocrinology*, **103**, 1742.

HORWITZ, K.B. & MCGUIRE, W.L. (1978). Estrogen control of progesterone receptor in human breast cancer. *J. Biol. Chem.*, **253**, 2223.

JOHNSON, M.D., WESTLEY, B.R. & MAY, F.E.B. (1989). Oestrogenic activity of tamoxifen and its metabolites on gene regulation and cell proliferation in MCF-7 breast cancer cells. *Br. J. Cancer*, **59**, 727.

KATZENELLENBOGEN, B.S., KENDRA, K.L., NORMAN, M.J. & BERTHOIS, Y. (1987). Proliferation, hormonal responsiveness, and estrogen receptor content of MCF-7 human breast cancer cells grown in the short-term and long-term absence of estrogens. *Cancer Res.*, **47**, 4355.

KAWASUMI, H., KAIBARA, N. & KOGA, S. (1988). Cocarcinogenic activity of bile acids in the chemical transformation of C3H/10T1/2 fibroblasts *in vitro*. *Oncology*, **45**, 192.

KIDA, N., YOSHIMURA, T., MORI, K. & HAYASHI, K. (1989). Hormonal regulation of synthesis and secretion of pS2 protein relevant to growth of human breast cancer cells (MCF-7). *Cancer Res.*, **49**, 3494.

KING, W.J. & GREENE, G.L. (1984). Monoclonal antibodies localise oestrogen receptor in the nuclei of target cells. *Nature*, **307**, 745.

KULKARNI, M.S., HEIDEPRIEM, P.M. & YIELDING, K.L. (1980). Production by lithocholic acid of DNA strand breaks in L1210 cells. *Cancer Res.*, **40**, 2666.

LAWRENCE, R.A., BAKER, P.R. & CUSCHIERI, A. (1980). Bile salt binding to hepatic ligandin and serum albumin of the rat. *Biochem. Soc. Trans.*, **8**, 372.

MAY, F.E.B. & WESTLEY, B.R. (1988). Identification and characterization of estrogen-regulated RNAs in human breast cancer cells. *J. Biol. Chem.*, **263**, 12901.

MAY, F.E.B., JOHNSON, M.D., WISEMAN, L.R., WAKELING, A.E., KASTNER, P. & WESTLEY, B.R. (1989). Regulation of progesterone receptor mRNA by oestradiol and antioestrogens in breast cancer cell lines. *J. Steroid Biochem.*, **33**, 1035.

MURRAY, W.R., BLACKWOOD, A., CALMAN, K.C. & MACKAY, C. (1980). Faecal bile acids and clostridia in patients with breast cancer. *Br. J. Cancer*, **42**, 856.

NARDULLI, A.M., GREENE, G.L., O'MALLEY, B.W. & KATZENELLENBOGEN, B.S. (1988). Regulation of progesterone receptor messenger ribonucleic acid and protein levels in MCF-7 cells by estradiol: analysis of estrogen's effect on progesterone receptor synthesis and degradation. *Endocrinology*, **122**, 935.

NUNEZ, A.-M., BERRY, M., IMLER, J.-L. & CHAMBON, P. (1989). The 5'-flanking region of the pS2 gene contains a complex enhancer region responsive to oestrogens, epidermal growth factor, a tumour promoter (TPA), the c-Ha-*ras* oncogene and the c-*jun* protein. *EMBO J.*, **8**, 823.

OELBERG, D.G., DOWNEY, S.A. & FLYNN, M.M. (1990). Bile salt-induced intracellular $Ca^{2+}$ accumulation in type II pneumocytes. *Lung*, **168**, 297.

OWEN, R.W., HENLY, P.J., THOMPSON, M.H. & HILL, M.J. (1986). Steroids and cancer: faecal bile acid screening for early detection of cancer risk. *J. Steroid Biochem.*, **24**, 391.

PAPATESTAS, A.E., PANVELLIWALLA, D., TARTTER, P.I., MILLER, S., PERSEMILIDIS, D. & AUFSES, A.H. (1982). Faecal steroid metabolites and breast cancer risk. *Cancer*, **49**, 1201.

RAJU, U., LEVITZ, M. & JAVITT, N.B. (1990). Bile acids in human breast cyst fluid: the identification of lithocholic acid. *J. Clin. Endocrinol. Metab.*, **70**, 1030.

READ, L.D., SNIDER, C.E., MILLER, J.S., GREENE, G.L. & KATZENELLENBOGEN, B.S. (1988). Ligand-modulated regulation of progesterone receptor messenger ribonucleic acid and protein in human breast cancer cell lines. *Molec. Endocrinol.*, **2**, 263.

SACEDA, M., LIPPMAN, M.E., CHAMBON, P. & 4 others (1988). Regulation of the estrogen receptor in MCF-7 cells by estradiol. *Mol. Endocrinol.*, **2**, 1157.

SUMIDA, C. & PASQUALINI, J.R. (1990). Stimulation of progesterone receptors by phorbol ester and cyclic AMP in fetal uterine cells in culture. *Molec. Cell. Endocrinol.*, **69**, 207.

WATABE, J. & BERNSTEIN, H. (1985). The mutagenicity of bile acids using a fluctuation test. *Mutation Res.*, **158**, 45.

WEI, L.L., KRETT, N.L., FRANCIS, M.D. & 4 others (1988). Multiple human progesterone receptor messenger ribonucleic acids and their autoregulation by progestin agonists and antagonists in breast cancer cells. *Molec. Endocrinol.*, **2**, 62.

WILTON, J.C., JONES, C.E., MILLS, C.O., ELIAS, E. & BAKER, P.R. (1990). Oestrogen-like activity of bile acid on steroid receptor levels of MCF-7 human breast cancer cells. (Abstract). *Br. J. Cancer*, **62** (Suppl XII), 29.